# Comparison of Short-Term Clinical Outcomes After Implantation of Two Monofocal, Aspheric Intraocular Lenses

**DOI:** 10.3390/diagnostics14242862

**Published:** 2024-12-19

**Authors:** Jeewon Han, Yea Eun Lee, Nahyun Park, Chung Min Lee, Yoo Young Jeon, Hayoung Lee, Kyu Sang Eah, Yeji Yoon, Ho Seok Chung, Jae Yong Kim, Jiwon Jeong, Hun Lee

**Affiliations:** 1 Department of Ophthalmology, Asan Medical Center, University of Ulsan College of Medicine, Seoul 05505, Republic of Korea; jenny4132@naver.com (J.H.); yeaeun812@gmail.com (Y.E.L.); laurenpark66@gmail.com (N.P.); chungminlee1215@gmail.com (C.M.L.); cheese_sauce@naver.com (Y.Y.J.); glory2822@naver.com (H.L.); kseah0124@gmail.com (K.S.E.); yejii0849@gmail.com (Y.Y.); chunghoseok@gmail.com (H.S.C.); jykim2311@amc.seoul.kr (J.Y.K.); 2 Department of Ophthalmology, Asan Medical Institute of Convergence Science and Technology, Asan Medical Center, University of Ulsan College of Medicine, Seoul 05505, Republic of Korea; 3 Department of Ophthalmology, Brain Korea 21 Project, University of Ulsan College of Medicine, Seoul 05505, Republic of Korea; 4 Fatima Vision Center, Changwon 51408, Republic of Korea; 5 Center for Cell Therapy, Asan Institute for Life Science, Seoul 05505, Republic of Korea

**Keywords:** CT LUCIA 621P IOL, contrast sensitivity, internal spherical aberration

## Abstract

Objectives: This study compared the visual outcomes and optical quality of two monofocal, aspheric intraocular lenses (IOLs; CT LUCIA 621P, Carl Zeiss Meditec; Eyhance ICB00, Johnson & Johnson Vision) by evaluating visual acuity, contrast sensitivity, and higher-order aberrations 1 month post-cataract surgery. Methods: In this retrospective, comparative study, 120 eyes (72 patients) that underwent cataract surgery with either CT LUCIA 621P (Lucia group) or Eyhance ICB00 (Eyhance group) implantation (60 eyes/group) were retrospectively investigated. Visual acuity at various distances and defocus curves were measured 1 month postoperatively. Optical quality was assessed by comparing contrast sensitivity and internal coma, spherical, and total aberrations by using iTrace (Tracey Technology), a ray-tracing-type aberrometer. Results: The visual acuity and defocus curves were similar between the two IOLs 1 month postoperatively. The Lucia group showed better contrast sensitivity at higher spatial frequencies: 12 cpd (*p* < 0.001, 1.32 LogCS vs. 1.02 LogCS) and 18 cpd (*p* = 0.009, 0.74 LogCS vs. 0.47 LogCS) unilaterally and 18 cpd (*p* = 0.044, 0.94 LogCS vs. 0.60 LogCS) bilaterally. Postoperative internal spherical aberration was significantly lower in the Lucia group (*p* < 0.001, −0.04 µm vs. −0.003 µm). Internal coma and total aberrations were similar. Conclusions: The visual acuity and defocus curves of the Lucia and Eyhance groups were comparable 1 month post-cataract surgery. The Lucia group’s superior contrast sensitivity and lower postoperative internal spherical aberration were due to differences in IOL designs, particularly the power variation patterns.

## 1. Introduction

Despite the popularity of multifocal intraocular lenses (IOLs), monofocal IOLs remain the most widely used in cataract surgery due to the clarity of vision at a selected distance, rare probabilities of photic phenomena, and relatively low cost [1]. With advances in optical technology over previous decades, various types of monofocal IOLs with different optical designs have been developed in an attempt to improve visual acuity after cataract surgery.

One of the strategies for the best postoperative optical performance includes the implantation of an aspheric IOL, designed to reduce the spherical aberration of the eye [2]. Previous studies have shown that the implantation of aspheric IOLs enhanced clarity of vision particularly by increasing contrast sensitivity, since they can eliminate corneal spherical aberration by utilizing a variable curvature in the lens surface [3,4,5]. Additionally, advances in monofocal IOLs have focused on not only improving optical clarity at the focal point, but also on widening the depth of focus, thereby providing better performance in daily activities after cataract surgery [1,6,7].

The Tecnis Eyhance^TM^ ICB00 (Johnson & Johnson Vision, Jacksonville, FL, USA) is a single-piece aspheric monofocal IOL, which was designed to induce a range of focus and thus enhance intermediate vision [1]. An increased depth of focus is achieved by a higher-order anterior surface of the lens with a continuous increase in power from the periphery to the center of the optic [2,6]. Several studies have reported enhanced intermediate visual performance, as well as similar distance performance and photic phenomena, using this IOL as compared with those using other monofocal IOLs [8,9,10,11,12,13].

The CT LUCIA**^®^** 621P Monofocal IOL (Carl Zeiss Meditec AG, Jena, Germany), a new single-piece hydrophobic acrylic aspheric IOL [4], features an optimized Zeiss-Optic (ZO) aspheric profile in which the dioptric power of the IOL is higher in the center and then varies toward the periphery, showing a non-constant aberration lens profile [4,14,15]. Additionally, the IOL’s architecture is equipped with a C-loop, step-vaulted haptic design, to maximize the stability when implanted in the capsular bag [16].

Although several studies have assessed the refractive and visual outcomes of the predecessor models of CT LUCIA, no studies to date have compared the clinical outcomes of the newest model, CT LUCIA 621P, and other enhanced monofocal IOLs, including the Tecnis Eyhance ICB00 IOL. Therefore, this study set out to compare the clinical outcomes and optical quality of these two different aspheric monofocal IOLs, the CT LUCIA 621P and the Eyhance ICB00, by investigating postoperative visual acuity at different distances, contrast sensitivity, and internal higher-order aberrations (HOAs) 1 month after uneventful cataract surgery.

## 2. Materials and Methods

This retrospective, comparative study was conducted in accordance with the tenets of the Declaration of Helsinki and was approved by the Public Institutional Review Board Designated by Ministry of Health and Welfare (file number: P01-202409-01-024). The need to obtain written informed consent from patients was waived due to the retrospective design of the study.

The medical records of patients who had undergone cataract surgery with implantation of the CT LUCIA 621P (Lucia group) or Eyhance ICB00 (Eyhance group) between January 2023 and June 2024 at a single center in South Korea were reviewed retrospectively. Patients aged 50 years or older, with clinically significant levels of cataract, and who had undergone standard phacoemulsification with implantation of either the CT LUCIA 621P or Eyhance ICB00 were included in the final analysis. For both unilateral and bilateral surgeries, IOLs targeting emmetropia were implanted. The exclusion criteria included patients with regular corneal astigmatism of >1.50 D or irregular corneal astigmatism, previous eye surgery or trauma, and other ocular diseases that could influence visual function after cataract surgery, such as glaucoma, intraocular inflammation, or retinopathy.

Preoperatively, all patients underwent comprehensive ophthalmic examinations. A detailed slit-lamp examination was conducted to evaluate the anterior segment, including the degree of cataract, using the Lens Opacification Classification System III (LOCS III). Keratometry was measured by using an auto kerato-refractometer (KR-800, Topcon, Tokyo, Japan), and intraocular pressure was measured by using a tonometer (CT80A, Topcon, Tokyo, Japan). Corneal tomography was evaluated using a Pentacam^®^ device (Oculus, Wetzlar, Germany), and biometry was performed with an IOLMaster 700 device (Carl Zeiss Meditec AG, Jena, Geramany). A detailed fundus examination was carried out with the pupil dilated to evaluate the retina and optic nerve.

All post-surgical visual acuity measurements were obtained 1 month after cataract surgery. Uncorrected distancevisual acuity (UDVA), corrected distance visual acuity (CDVA), uncorrected intermediate visual acuity (UIVA) at 80 cm and 60 cm, and uncorrected near vision acuity (UNVA) were measured by using a logarithmic visual acuity chart (ETDRS; Precision Vision, Woodstock, IL, USA). Defocus curves were obtained both monocularly and binocularly 1 month after surgery using defocusing lenses with a power range between 1.00 D and −3.50 D, in 0.5-D increments. The measurement was carried out using ETDRS charts under both photopic and mesopic light conditions. Contrast sensitivity was evaluated using a Functional Visual Analyzer (STEREO OPTICAL Company Inc. Chicago, IL, USA). Internal aberrations, including coma, spherical, and total aberrations, were assessed using ray-tracing aberrometry (iTrace, Tracey Technology, Houston, TX, USA).

### 2.1. Surgical Procedures

All cataract surgical procedures were performed under topical anesthesia using the Centurion Silver Vision System (Alcon Laboratories, Inc., Fort Worth, TX, USA). The main corneal incision was made by a 2.2 mm blade at the temporal side. An anterior capsular opening of 5.0 mm was created with continuous curvilinear capsulorhexis. After the phacoemulsification process, the IOL was implanted and centered within the capsular bag. All corneal wounds were sealed with stromal hydration. Postoperatively, all patients were administered 1% prednisolone acetate suspension (Pred Forte^®^; Allergan, Inc., Irvine, CA, USA) and 0.5% moxifloxacin (Vigamox^®^; Alcon Laboratories, Inc., Forth Worth, TX, USA) for 1 month.

### 2.2. Statistical Analysis

IBM SPSS Statistics for Windows (25.0 version, IBM Corp., Armonk, NY, USA) was used to perform the statistical analysis. Kolmogorov–Smirnov tests were performed to check the normality of the data distribution. Then, independent t-tests and Mann–Whitney U tests were used to examine the differences between the two groups. *p*  <  0.05 was considered statistically significant.

## 3. Results

Overall, 120 eyes from 72 patients were enrolled in this study, with 60 eyes (36 patients) implanted with the CT LUCIA 621P IOL (Lucia group) and 60 eyes (36 patients) implanted with the Eyhance ICB00 IOL (Eyhance group). The demographic characteristics of the patients, including age, sex, preoperative and postoperative UDVA, CDVA, spherical power, cylindrical diopter, and spherical equivalent, are summarized in Table 1. No statistically significant differences were seen between the two groups in terms of baseline characteristics.

**Figure 1:**
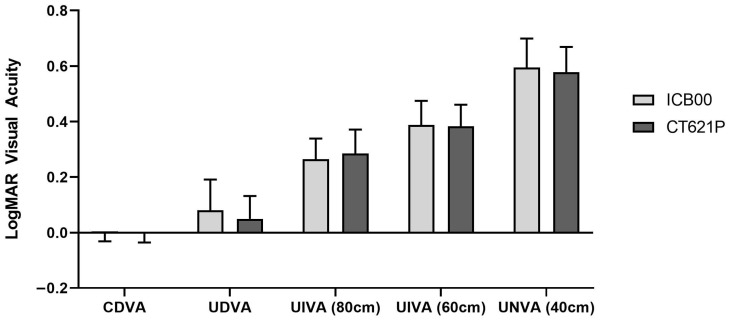
Comparison of visual acuity between the two groups measured at different distances. CDVA: corrected distance visual acuity; UDVA: uncorrected distance visual acuity; UIVA: uncorrected intermediate visual acuity; UNVA: uncorrected near visual acuity.

The postoperative corrected visual acuity at a far distance and the uncorrected visual acuity outcomes at near (40 cm), intermediate (60 cm and 80 cm), and far (4 m) distances were compared between the two groups (Figure 1). The Lucia group demonstrated comparable CDVA, UDVA, and UNVA to the Eyhance group (Table 1 and Table 2).

**Figure 2:**
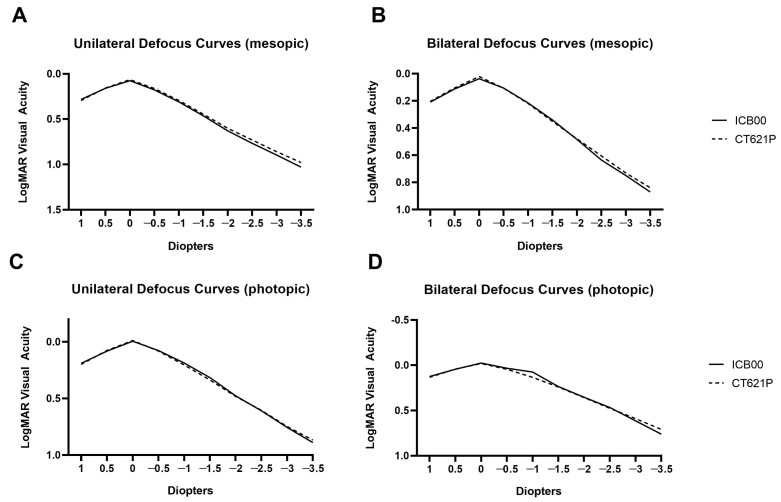
(**A**) Unilateral and (**B**) bilateral defocus curves showing logMAR visual acuity at different distances measured under mesopic conditions. (**C**) Unilateral and (**D**) bilateral defocus curves showing logMAR visual acuity at different distances measured under photopic conditions.

Defocus curves were generated for both unilateral and bilateral eyes under both photopic and mesopic conditions, covering a range from +1.0 D to −3.5 D (Figure 2). Both groups showed comparable visual performance across a broad range of defocus values, making the IOLs similarly appropriate in terms of providing consistent vision at various distances.

**Figure 3:**
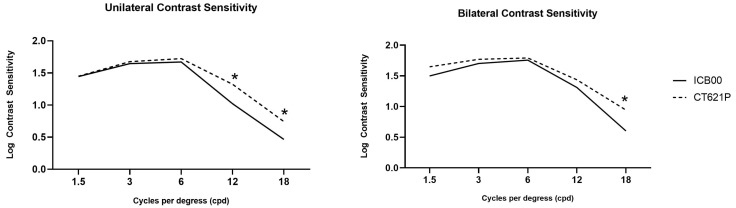
Unilateral and bilateral mean contrast sensitivity values under photopic condition for two groups. * *p* < 0.05.

Figure 3 demonstrates the results of mean contrast sensitivity for both groups. The Lucia group demonstrated superior contrast sensitivity at higher spatial frequencies, with statistically significant differences at 12 and 18 cpd in unilateral eyes (*p* < 0.001 and *p* = 0.009, respectively) and at 18 cpd in bilateral eyes (*p* = 0.044). These findings suggested that the CT LUCIA 621P IOL could provide better overall contrast sensitivity, which may contribute to enhanced visual performance in low-contrast environments.

**Figure 4:**
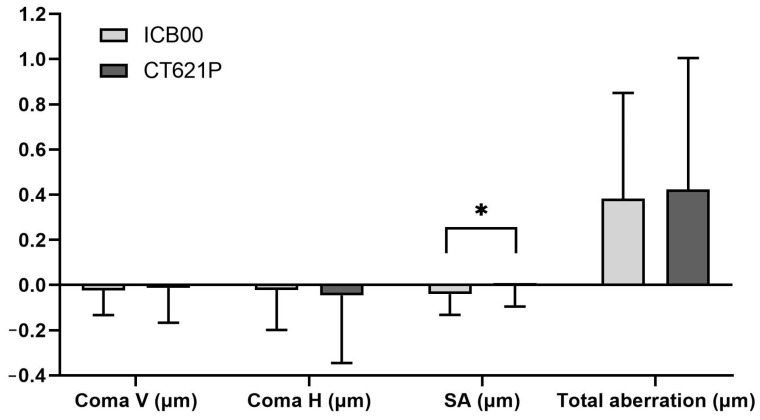
Results of iTrace measurements. Internal aberrations, including coma, spherical, and total aberrations, measured in both groups. Coma V: internal coma, vertical; Coma H: internal coma, horizontal; SA: internal spherical aberration; Total aberration: total internal aberration. * *p* < 0.001.

Preoperatively, there were no statistically significant differences between the two groups in any parameters of aberration measured using iTrace. Table 3 and Figure 4 demonstrate the postoperative aberrometry measurements, including pupil diameter, scan size, and coma, spherical, and total aberrations obtained using the iTrace system (Tracey Technology). Both groups had similar pupil diameters and scan sizes at the postoperative measurement. The CT LUCIA 621P IOL resulted in significantly lower levels of internal spherical aberration than did the Eyhance IOL (*p* < 0.001; −0.04 μm for the Eyhance IOL and −0.003 μm for the CT LUCIA 621P IOL).

## 4. Discussion

In the present study, we compared the postoperative visual acuity, contrast sensitivity, and HOAs between the CT LUCIA 621P, a standard monofocal IOL, and the Tecnis Eyhance ICB00, an enhanced monofocal IOL. Both IOLs yielded comparable visual outcomes; however, the Lucia group exhibited superior contrast sensitivity at higher spatial frequencies under photopic conditions, along with significantly lower internal spherical aberration values.

In terms of visual outcomes, both groups showed excellent distant visual acuity 1 month after surgery. The mean postoperative UDVA and CDVA were 0.08 ± 0.11 and 0.00 ± 0.03 logMAR in the Eyhance group and 0.05 ± 0.08 and 0.00 ± 0.03 logMAR in the Lucia group. Good visual outcomes of the Eyhance ICB00 have been reported in various studies, in which the postoperative distant visual acuity results were similar to the results of our study [1,15,17]. As the CT LUCIA 621P is relatively novel, only a limited number of studies have investigated its visual performance to date [4,16]. In a retrospective study by Hernández-Martínez et al., a 1-month postoperative Snellen CDVA of 20/25 or better was reported in 80% of eyes implanted with a CT LUCIA 621P IOL, and the change in the lines of visual acuity between the postoperative UDVA and CDVA was either absent or improved [16]. In another study, a cumulative Snellen visual acuity of 20/25 or better was observed in 100% of eyes implanted with a CT LUCIA 621P 3 months postoperatively [4]. These favorable visual outcomes are in line with the results of our study, in which 91.6% of the eyes implanted with the CT LUCIA 621P IOL achieved a Snellen CDVA of 20/25 or better.

The uncorrected visual acuity at intermediate and near distances was comparable between the two groups. The mean postoperative UIVAs were 0.39 ± 0.09 and 0.26 ± 0.07 logMAR in the Eyhance group and 0.38 ± 0.08 and 0.28 ± 0.09 logMAR in the Lucia group, at 60 and 80 cm, respectively. Although the Eyhance group exhibited better intermediate vision at a distance of 80 cm, the difference between the two groups was not statistically significant. An advantage of the Eyhance IOL in terms of better postoperative intermediate vision has been reported in previous studies, with the monocular UIVA ranging from 0.24 to 0.31 logMAR [9,17,18] and the distance-corrected intermediate visual acuity (DCIVA) ranging from 0.01 to 0.20 logMAR [9,17,19,20].

The monocular and binocular defocus curves showed comparable results in both groups under photopic and mesopic light conditions. Although the photopic binocular defocus curve of the Eyhance group was wider between 0.0 D and −1.0 D, seemingly generating an extended focal plane, the difference between the two groups was not statistically significant at any of the examined distances. In previous studies, the defocus curve of the Eyhance ICB00 was smoother, with a wider landing zone, than that of other monofocal IOLs, providing significantly better visual acuity, particularly within the intermediate defocus range up to −1.50 D [1,2,8,9,10,11,21].

**Figure 5:**
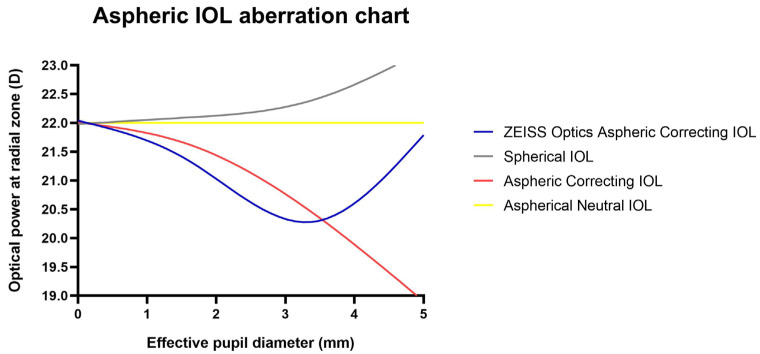
Asphericity changes in different types of IOLs across pupil diameters. Courtesy of Zeiss Meditec (Jena, Germany). ZEISS Optics (ZO) aspheric correcting IOLs feature a non-constant aberration profile, with negative spherical aberration in the central zone transitioning gradually to positive spherical aberration in the periphery, resulting in an aberration-neutral effect. IOL = intraocular lens; D = diopters.

The comparable visual outcomes in both groups can be attributed to the differences in aspheric IOL designs, particularly their power variation patterns (Figure 5) [14]. The Eyhance IOL improved intermediate vision through its unique anterior surface, which is specifically designed to produce a continuous power change from the periphery to the center [20]. The CT LUCIA 621P was designed with a ZO aspheric concept optic, known as non-constant aberration [14]. Specifically, the central optic zone (~3.5 mm) of the CT LUCIA 621P was designed with negative spherical aberration to counteract the positive aberration of the natural cornea. According to the manufacturer, the extent to which the ZO optic corrects spherical aberration varies with pupil diameter: −0.24 µm at 3 mm, −0.16 µm at 4.27 mm, and −0.08 µm at 5 mm. The negative spherical aberration in the central optic then gradually transitions towards positive spherical aberration in the peripheral zone [14]. This transition was initially intended to improve decentration forgiveness; however, it also extends the depth of focus due to the residual spherical aberration from the peripheral aberration-neutral zone of the optic [22]. Therefore, these IOL types seem to yield comparable visual outcomes.

Other biometric factors, such as pupil size, could also have affected the visual outcomes. A prospective study by Micheletti et al. reported that patients with significantly enhanced intermediate vision (DCIVA ≤ 0.2 logMAR) after implantation of the Eyhance ICB00 showed a smaller mean pupil size (3.64 mm), measured under mesopic conditions by iTrace, suggesting that pupil size could be a predictor of increased intermediate vision [23]. Moreover, a retrospective case–control study found that the Eyhance ICB00 demonstrated optimal intermediate performance at a 2.0 mm pupil size when tested across various artificial pupil sizes. [20] This result was consistent with that of another in vitro study that showed better optical performance of the Eyhance IOL with small pupil sizes (2.0–3.5 mm) [24]. Given that the mean pupil sizes measured under mesopic condition by iTrace in our study were 3.94 mm and 3.92 mm for the Eyhance and Lucia groups, respectively, the relatively larger pupil size in our study may have impeded the Eyhance IOL’s ability to utilize its extended depth of focus fully. Further research is required to explore the extent to which the visual outcomes of these two IOLs vary with changes in pupil size.

The Lucia group provided superior contrast sensitivity under photopic conditions at 12 cpd and 18 cpd in unilateral eyes and at 18 cpd in bilateral eyes. These findings align with those of previous studies comparing the Eyhance ICB00 with other monofocal IOLs. The Eyhance ICB00, designed as an enhanced monofocal IOL, can offer improved depth of focus without significantly compromising contrast sensitivity at lower spatial frequencies [1,15,17,19]. However, it tended to show a slight decline in contrast sensitivity at higher spatial frequencies in previous studies [1,10,12]. The better performance of the Lucia group at higher spatial frequencies, as demonstrated in this study, can be attributed to its aspheric optic design. The optic of the Lucia IOL is optimized for corneal asphericity and is designed to compensate for a range of aberrations, which may result in limited light scattering and improved contrast sensitivity with higher image quality [4,14]. This enhanced performance, particularly at higher spatial frequencies, suggests that the CT LUCIA 621P may provide better visual outcomes in demanding environments, such as during night driving or when reading fine print [25]. These findings highlight that the CT LUCIA 621P may be a preferable choice for those prioritizing visual clarity and contrast sensitivity in daily activities.

When evaluating HOAs, we specifically focused on internal HOAs arising from internal optics, including vertical and horizontal coma, spherical aberration, and total HOAs [22]. While spherical aberration was significantly lower in the Lucia group, we found no significant differences between the two groups for the other parameters. This outcome is consistent with the results from previous research, showing that the Eyhance IOL maintains similar coma and total aberration profiles to those of other monofocal IOLs [26]. The findings from another study comparing the CT LUCIA 611P and Tecnis-1 ZCB00 IOLs further corroborated the results of our study [22]. In that study, the Tecnis-1 ZCB00 IOL group had higher internal coma and spherical aberration, although the total HOAs and coma and spherical aberration values were comparable between the two IOLs [22]. The authors indicated that the Tecnis-1 ZCB00 IOL was expected to have higher internal spherical aberrations than the CT LUCIA 611P IOL (−0.12 μm) due to the greater negative spherical aberration of the optic (−0.27 μm). Similarly, in our study, the Eyhance IOL showed higher internal spherical aberration (−0.04 μm) than did the CT LUCIA 621P IOL (−0.003 μm), consistent with the findings of the previous study.

The non-constant aberration aspheric design of the LUCIA model, which features a flatter surface at intermediate distances and a steeper curvature towards the lens periphery, has been proposed to help accommodate various corneal shapes and could potentially reduce the incidence of HOAs [14,22]. While this design possibly contributed to reducing internal spherical aberration in our study, it did not significantly reduce overall internal aberrations, indicating that other factors, such as coma or trefoil, should also be considered when evaluating total aberrations. The comparable internal total HOAs between the two groups further supported the equivalent visual performance of the CT LUCIA 621P and Eyhance ICB00, as reflected in the nearly identical defocus curves.

This study had some limitations. The follow-up period in this study was relatively short. Longer-term follow-up is necessary to assess the stability of visual outcomes, the development of posterior capsule opacification, and the long-term impact of IOL design on visual quality, including its tolerance to tilt and decentration. Since pupil size can influence the visual function of IOLs, further investigation into how varying pupil sizes affect the performance of these IOLs, particularly under different lighting conditions, could provide a more comprehensive understanding of these two IOLs. Lastly, this study primarily focused on visual acuity and contrast sensitivity but did not thoroughly explore other visual functions, such as halo and glare sensitivity or patient-reported outcomes. Further studies that include these factors are warranted.

## 5. Conclusions

Both IOLs demonstrated competent visual acuity at all distances, with the Eyhance ICB00 showing a slight advantage in binocular intermediate vision. The CT LUCIA 621P provided superior contrast sensitivity, particularly at higher spatial frequencies, and lower internal spherical aberration, probably due to its proprietary aspheric optic design. Therefore, the CT LUCIA 621P may be a better choice for those prioritizing contrast sensitivity and visual clarity under demanding conditions.

## Figures and Tables

**Table 1:** Patient demographics.

	IOL		
Parameter	Eyhance (J&J, ICB00) (*n* = 60)	Lucia (Zeiss, CT 621P) (*n* = 60)	*p* Value
Age (years)	69.26 ± 5.83 (55 to 79)	68.39 ± 4.48 (59 to 77)	0.277
Sex (male/female)	16 (44.4%)/20 (55.6%)	19 (52.8%)/17 (47.2%)	0.737
UDVA (logMAR)			
Preoperative	0.52 ± 0.34 (0.05 to 1.70)	0.51 ± 0.34 (0.10 to 1.52)	0.823
Postoperative	0.08 ± 0.11 (−0.08 to 0.52)	0.05 ± 0.08 (−0.08 to 0.40)	0.069
CDVA (logMAR)			
Preoperative	0.25 ± 0.28 (0.00 to 1.70)	0.25 ± 0.33 (0.00 to 1.52)	0.533
Postoperative	0.00 ± 0.03 (−0.08 to 0.05)	0.00 ± 0.03 (−0.08 to 0.10)	0.681
Spherical power (D)			
Preoperative	0.57 ± 1.37 (−3.75 to 3.25)	0.41 ± 1.60 (−4.40 to 3.00)	0.696
Postoperative	−0.15 ± 0.35 (−1.00 to 0.75)	−0.09 ± 0.31 (−1.00 to 0.50)	0.265
Cylindrical diopter (D)			
Preoperative	−0.68 ± 0.57 (−1.50 to 0.00)	−0.68 ± 0.54 (−1.50 to 0.00)	0.932
Postoperative	−0.33 ± 0.36 (−1.25 to 0.00)	−0.38 ± 0.40 (−1.50 to 0.00)	0.709
Spherical equivalent (D)			
Preoperative	0.18 ± 1.39 (−4.13 to 2.75)	0.06 ± 1.56 (−4.00 to 2.88)	0.744
Postoperative	−0.31 ± 0.32 (−1.00 to 0.25)	−0.29 ± 0.34 (−1.00 to 0.38)	0.590

Values are presented as mean ± standard deviation (range) or number of eyes (percentage). IOL: intraocular lens; UDVA: uncorrected distance visual acuity; CDVA: corrected distance visual acuity; D: diopters. Chi-square test was used for sex, and Mann–Whitney U test was used for age, UDVA, CDVA, spherical power, cylindrical diopter, and spherical equivalent.

**Table 2:** Postoperative UIVA (80 cm, 60 cm) and UNVA (40 cm).

	IOL		
Parameter	Eyhance (J&J, ICB00) (*n* = 60)	Lucia (Zeiss, CT 621P) (*n* = 60)	*p* Value
UIVA (80 cm)	0.26 ± 0.07 (0.10–0.40)	0.28 ± 0.09 (0.10–0.49)	0.229
UIVA (60 cm)	0.39 ± 0.09 (0.20–0.49)	0.38 ± 0.08 (0.20–0.60)	0.548
UNVA (40 cm)	0.60 ± 0.10 (0.40–0.80)	0.58 ± 0.09 (0.40–0.80)	0.631

Values are presented as mean ± standard deviation (range). IOL: intraocular lens; UIVA (80 cm): uncorrected intermediate visual acuity measured at 80 cm; UIVA (60 cm): uncorrected intermediate visual acuity measured at 60 cm; UNVA (40 cm): uncorrected near visual acuity measured at 40 cm. Mann–Whitney U test was used.

**Table 3:** Postoperative pupil diameters and internal higher-order aberrations.

	Intraocular lens		
Parameter	Eyhance (J&J, ICB00) (*n* = 60)	Lucia (Zeiss, CT 621P) (*n* = 60)	*p* Value
Pupil diameter (mm)	3.94 ± 0.76 (2.60 to 5.91)	3.92 ± 0.70 (2.57 to 5.40)	0.977
Scan size (mm)	3.41 ± 0.43 (2.00 to 4.30)	3.50 ± 0.37 (2.50 to 4.40)	0.323
Vertical coma (μm)	−0.02 ± 0.11 (−0.28 to 0.25)	−0.01 ± 0.15 (−0.22 to 0.81)	0.830
Horizontal coma (μm)	−0.02 ± 0.18 (−0.91 to 0.59)	−0.05 ± 0.30 (−1.82 to 0.28)	0.645
Spherical aberration (μm)	−0.04 ± 0.09 (−0.23 to 0.22)	−0.003 ± 0.09 (−0.24 to 0.41)	<0.001
Total aberration (μm)	0.38 ± 0.47 (0.07 to 2.58)	0.42 ± 0.58 (0.05 to 3.66)	0.547

Values are presented as mean ± standard deviation (range). Mann–Whitney U test was used.

## Data Availability

The datasets generated and/or analyzed during the current study are available from the corresponding author on reasonable request.
